# Are school-level factors associated with primary school students' experience of physical violence from school staff in Uganda?

**DOI:** 10.1093/inthealth/ihv069

**Published:** 2015-12-08

**Authors:** Louise Knight, Janet Nakuti, Elizabeth Allen, Katherine R. Gannett, Dipak Naker, Karen M. Devries

**Affiliations:** a London School of Hygiene and Tropical Medicine, 15–17 Tavistock Place, London, UK; b Raising Voices, 16 Tufnell Drive, Kamwokya P.O. Box 6770, Kampala, Uganda

**Keywords:** Multilevel logistic regression, Schools, School connectedness, Uganda, Variance partition coefficient, Violence

## Abstract

**Background:**

The nature and structure of the school environment has the potential to shape children's health and well being. Few studies have explored the importance of school-level factors in explaining a child's likelihood of experiencing violence from school staff, particularly in low-resource settings such as Uganda.

**Methods:**

To quantify to what extent a student's risk of violence is determined by school-level factors we fitted multilevel logistic regression models to investigate associations and present between-school variance partition coefficients. School structural factors, academic and supportive environment are explored.

**Results:**

53% of students reported physical violence from staff. Only 6% of variation in students' experience of violence was due to differences between schools and half the variation was explained by the school-level factors modelled. Schools with a higher proportion of girls are associated with increased odds of physical violence from staff. Students in schools with a high level of student perceptions of school connectedness have a 36% reduced odds of experiencing physical violence from staff, but no other school-level factor was significantly associated.

**Conclusion:**

Our findings suggest that physical violence by school staff is widespread across different types of schools in this setting, but interventions that improve students' school connectedness should be considered.

## Introduction

School-aged children globally spend more time in school than in any other single location besides the family home;^1^ therefore, the nature and structure of the school environment has a potential to have a great impact on children's health and wellbeing.^2^ Recent national prevalence studies in Kenya and Tanzania have shown that children report more exposure to violence from school staff than from parents, with 40% of 13–17 year olds in Kenya reporting being punched, kicked or whipped by a teacher in the last week and 13–15% reporting the same from parents. In Tanzania, 50% of respondents reported experiencing physical violence from a teacher when they were under 18 years of age.^3,4^

Understanding what predicts children's exposure to violence from school staff is necessary to address this form of violence against children. Some children are more likely to experience violence than others:^5–7^ a child's age, poor mental health and experience of violence from those besides school staff are associated with increased risk of violence from school staff in Uganda.^8^ However, little research has considered school-level factors and how these might relate to a child's risk of violence, particularly in low-resourced settings in Africa, including Uganda.^9,10^

In other fields, school-level factors are important determinants of child outcomes. In relation to educational achievement, school-level factors have been shown to explain up to 20% of variation in children's outcomes in the UK^11,12^ and 64% in South Africa.^13^ A recent literature review of multilevel models exploring school-level factors in relation to health outcomes, including whether a school is public or private, school size, class size and pupil-to-teacher ratio, reported mixed findings from mainly high income settings,^10^ and there is some evidence that school-level factors are associated with later health outcomes in British adults.^14^

**Figure 1.**
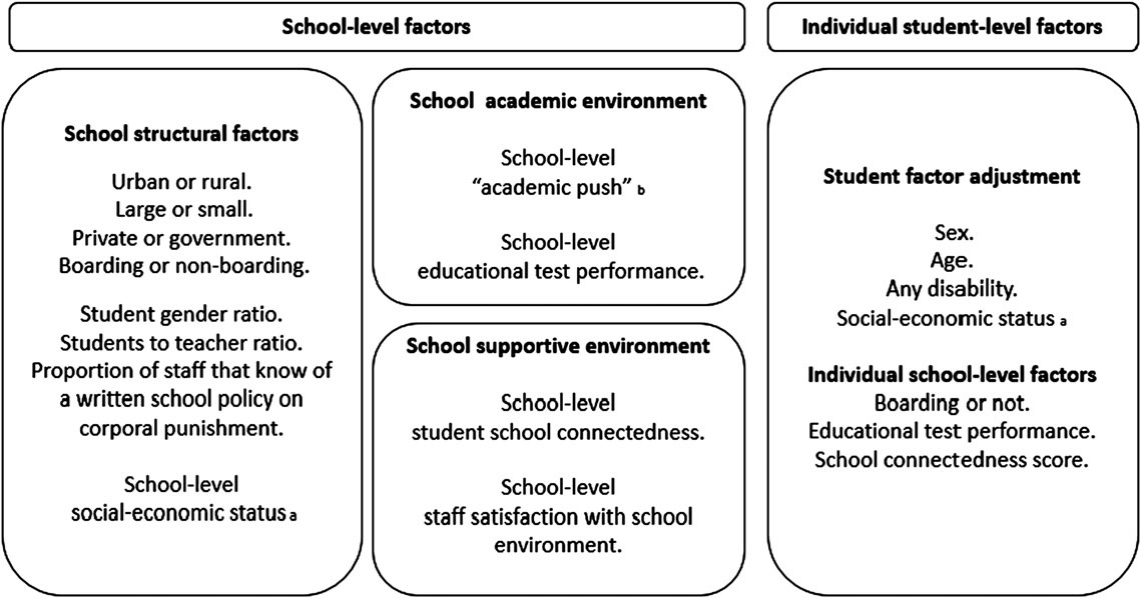
Summary of school-level factors explored for possible association with physical violence from staff in Ugandan schools and the student-level factors included in models.

^a^Social-economic level: number of hours student works outside of school, students report one or more adults sleeping in same room or not, students report two or more children sleeping in same room or not, and students report three or more meals eaten yesterday. School-level: aggregates of students reports.

^b^Schools known for their academic push were identified by one Raising Voices programme monitoring officer, prior to analysis. These schools were observed to be driven by a focus on exam result attainment above that of the other schools.

Figure 1 describes school-level factors that we hypothesised to be associated with a student's increased risk of exposure to violence from school staff. We hypothesised that students would report higher exposure to violence in schools that are located in rural areas; larger; private; boarding; higher student-to-teacher ratio; higher girl-to-boy ratio; less staff have knowledge of a school corporal punishment policy; and where the student population is of lower socioeconomic status.

Urban schools may be better resourced and may be more likely exposed to information about alternative discipline techniques. Boarding schools may be different in organisational environment, and students spend more time at boarding school and, therefore, have more opportunity to be exposed to violence. More academically focused schools are anecdotally seen as using more discipline to ‘push’ students to achieve high grades. Qualitative findings from Uganda suggest common perceptions that physical violence is necessary to discipline and guide children to appropriate behaviour, including high achievement in school.^15^ Private schools may be more resourced than government funded, yet more likely to ‘push’ students academically. However, in high-resourced settings, there is some evidence that schools that add value educationally may promote better health.^9^

Previous work has shown an association between low socioeconomic status and increased risk of violence.^5,6,16,17^ Schools where students are of low socioeconomic status may have poorer physical infrastructure and fewer teaching provisions (e.g. books). Low socioeconomic status, larger schools and higher student-to-teacher ratios may lead to strained teaching environments, frustration and use of violence by staff to maintain control in and out of the classroom. We hypothesised that a higher proportion of girls enrolled in school might be an important school-level factor, given that girls report more violence from staff than boys in this study population. We predicted that students in schools where there is a supportive environment for students and staff would report lower levels of violence because there would be less harsh discipline as a result of staff frustration because they feel more supported, motivated and happier in their work. In addition, students may behave better in an environment that is conducive to learning, with better communication and relationships between teachers and students. Student school connectedness has been shown to be an important protective factor against a range of negative health and wellbeing behaviours in older American adolescents, including, violence, substance misuse and school absenteeism.^2,18^

This study sought to quantify to what extent a student's risk of violence is determined by school differences and to investigate associations between school-level factors and student exposure to physical violence from school staff. Specific objectives were to: 1. describe the school-level factors of the study schools, 2. explore to what extent a student's risk of violence is determined by what school they attend, and 3. identify any significant associations between school-level factors investigated and level of physical violence from school staff reported by students, and to describe the amount of between-school variance explained by these factors.

## Methods

### Study design and data collection

We used baseline cross-sectional survey data from the Good Schools Study: a cluster randomised controlled trial of the Good School Toolkit to reduce violence against children in schools. Study methods and student characteristics have been fully described elsewhere.^8,19–21^ The baseline survey was conducted in primary schools in Luwero district, Uganda, in June 2012. As a large district with urban trading centres and rural sub-districts, Luwero was selected to serve as a typical Ugandan district, although it does not represent the capital city of Kampala. Using school enrolment lists, 151 eligible primary schools were identified and grouped into one of three strata according to the sex ratio of their students (>60% girls, >60% boys or approximately even). Forty-two primary schools were randomly selected proportional to the stratum size and all agreed to participate. Within these 42 schools, 3706 students in grades 5, 6 and 7 were interviewed—a response rate of 77%. In addition to the student survey, we incorporated data from a baseline survey conducted with school staff at the 42 schools (also in June 2012). All staff at each school were invited and 577 agreed to participate: consisting of 6% head teachers, 76% other teachers, 5% administrators and 13% other support staff (of which the majority were cooks who alone made up 6% of the total sample). Parents were informed of the survey and were given the option to opt-out their child; headmasters, students and staff provided individual consent to participate.

### Outcome measure

Students' self-reports of physical violence from staff in the past week were measured using items adapted from the International Society for the Prevention of Child Abuse and Neglect Child Abuse Screening Tool-Child Institutional (ICAST-CI; see Supplementary appendix).^22^

### Student-level adjustment

Our main aim was to explore the relationship between school-level factors and students' experience of violence from school staff. Student-level confounders were associated with one or more of the school-level factors and associated with physical violence from school staff. These included student's age, modelled as a continuous variable; sex, disability and socioeconomic status, measured by whether or not three meals or more were eaten in the previous day, whether the student shared a sleeping space with two or more other children, if student shared sleeping space with one or more adults modelled as binary variables; and the number of hours the student worked out of school (paid or unpaid) modelled in three categories: less than 1 hour, 1–2 hours and over 2 hours. In addition, relevant models were adjusted for whether a student was currently boarding, educational test performance and school connectedness, given the student-level measure association with the corresponding school-level factors of interest.^23^

A score for school connectedness (ranging from 0–12) was obtained by summing student responses to four questions (Supplementary appendix); Cronbach's alpha: 0.71. Individual educational test measures were adapted from a trial in Kenya and the Ugandan Early Grade Reading Assessments.^24^ A global educational performance score relative to peers was computed by summing the number of times a student scored in the bottom third of the overall distribution for each individual educational test and dividing by the number of completed tests. Those in the bottom 10% of students from this distribution were coded as ‘low performers’.^8^

### School-level measures

School-level socioeconomic measures were generated by aggregating non-boarding student data in to four student-level variables: whether or not three meals or more were eaten in the previous day, whether the student shared a sleeping space with two or more other children, if the student shared sleeping space with one or more adults and number of hours child worked outside of school (paid or unpaid). Mean scores were derived and dichotomised to above and below the median school-level score for each variable. Each resulting variable was modelled as binary. We investigated a composite measure to represent school-level socioeconomic status; the combined Cronbach's alpha was low (0.56) and, thus, the three school-level socioeconomic measures were modelled separately.

Schools were categorised by gender ratio (>60% boys, >60 girls and approximately even; government statistics); average number of students per teacher in classroom, calculated from classroom observations once per term for the four school terms following the baseline survey and modelled in three equal groups of 14 schools, small, median and large number of students per teacher; and proportion of school staff who knew of a written school policy on corporal punishment was modelled as a continuous variable. A school mean score for low educational test performance was derived by dividing the total number of low performers by the total number of students sampled. Subsequently, schools were categorised as having ‘low education performance’ if over 15% of students were low performers. Schools known for their academic push were identified by a programme monitoring officer from Raising Voices, the non-governmental organisation implementing the intervention, prior to analysis. These schools were observed to be driven by a focus on exam result attainment above that of the other schools.

Two variables measured school supportive environment: one by aggregating responses from students school connectedness scores by school and the other measured staff satisfaction with school environment by summing responses to 14 questions (Supplementary Appendix; Cronbach's alpha: 0.73). Both school supportive environment measures were modelled as binary variables, with school means dichotomised at above or below 50%, to represent schools with higher and lower overall student and staff supportive environment.

### Analysis

All analyses were conducted using Stata 13 (StataCorp, College Station, TX, USA). First, school factors were summarised with means, SDs and range for continuous variables and number of schools and percentages presented for discrete variables (Table 1).

**Table 1.** Summary of school-level factors

	Schools mean (SD), range	Number of schools (%)
Number of schools	42	42
School structural factors		
Urban	NA	15 (35.7)
Large^a^	NA	27 (64.3)
Private	NA	4 (9.5)
Boarding	NA	8 (19.1)
School male to female student ratio		
Approximately equal	NA	35 (83.3)
>60% girls	NA	4 (9.5)
>60% boys	NA	3 (7.1)
Proportion of staff that know of a written school policy on corporal punishment	0.4 (0.2), 0–0.8	NA
Average number of students per teacher in class^b^	42.4 (19.7), 12.7–92.3	NA
School socioeconomic status^c^		
Children had three or more meals yesterday	0.5 (0.1), 0.2–0.7	NA
Children shared sleeping area with two or more other children	0.6 (0.1), 0.4–0.8	NA
Children shared sleeping space with one or more adults	0.3 (0.1), 0.2–0.5	NA
Number of hours children working outside of school (paid or unpaid)^d^	1.8 (0.2), 1.3–2.3	NA
School academic environment		
High ‘academic push’ schools	NA	19 (45.2)
Low educational tests performing schools^e^	0.1 (0.1), 0–0.4	14 (33.3)
School supportive environment^f^		
Students school connectedness	9.3 (0.5), 8.1–10.5	NA
Staff satisfaction with school environment	24.3 (2.7), 18.6–29.4	NA

NA: not applicable.

^a^ Large: schools with over 84 students—based on sampled children.

^b^ Sum of the four observed class sizes divided by four teachers (1 teacher per class).

^c^ School means were dichotomised into high or low at 50%; therefore, approximately 21 schools in high and low categories.

^d^ Number of hours worked outside school for individual students scored: 1 for <1 hour, 2 for 1–2 hours and 3 for >2 hours. School mean dichotomised into high or low at 50%; therefore, approximately 21 schools in high and low categories.

^e^ Schools with over 15% of students categorised as performing low on educational tests.

^f^ School means were dichotomised into high or low at 50%; therefore, approximately 21 schools in high and low categories.

Second, to estimate to what extent a student's risk of violence is determined by school differences a multilevel mixed-effects logistic regression model was fitted with students at level one and school at level two. A null model was fitted for the binary outcome physical violence from staff in the past week and the Variance Partition Coefficient (VPC) was calculated to estimate the between-school variance using the latent variable method, formula shown in Supplementary Appendix.^25–29^ The VPC is a measure of intra-class correlation coefficient (ICC); that is, the proportion of total violence outcome variance that is associated with the school-level. There are a number of methods to estimate VPC; we chose to use the latent variable method as it is independent of prevalence and appropriate if the binary response is based on an underlying continuum, which is the case of our violence outcome.^25,26^

Third, to test our hypotheses that one or more school-level factors have an effect on violence experienced by students in school, a series of multilevel logistic regression models were fitted.

Additionally, the general effect of each model was calculated, including the proportional change in variance from the previous model with less terms (formula shown in Supplementary Appendix) and the VPC to quantify the between-school variance of physical violence explained by the school-level factors investigated.

No cross-level interactions were hypothesised. An interaction had been previously identified between students' performance in education tests and gender;^8^ this was not hypothesised to effect the school-level associations explored in this analysis. However, sensitively analysis was conducted with the interaction term included in the final model with no significant difference in presented results for all school-level factors.

## Results

The prevalence of past week physical violence from school staff, self-reported by students (the majority aged between 11 and 14 years; 81%) was 1990/3706 (53.7%).

### School-level factors

School-level factors are described in Table 1. In total, 15 schools were urban and 27 were classified as large. All urban schools were large and included four private boarding schools.

### Between-school variance in student reports of physical violence from staff

Our null model indicates that 6.19% of variation in a student's likelihood of experiencing physical violence from school staff is due to school-level factors, with strong evidence of significant between-school variance (likelihood ratio statistic p<0.001).

### Associations between individual factors and reported violence exposure

Individual level factors that were associated with increased physical violence in the past week included being female, younger age and working for more hours outside of school. There is some indication that children who have consumed a greater number of meals yesterday are at less risk of violence, although this finding is of borderline statistical significance.

### Associations between school factors and reported violence exposure

Findings suggest that after adjusting for individual factors, none of the school structural factors—urban or rural, private or government, large or small, boarding school or not, student teacher ratio, student gender ratio, or school-level social economic measure—were significantly associated with student reports of physical violence from staff in the last week. When controlling for student and structural factors, none of the other academic environment factors were significantly associated with physical violence experienced by students in those schools. The final model was additionally fitted with school supportive environment measures. Students in schools with higher levels of student connectedness had 36% lower odds of reporting violence from school staff. Schools with a higher proportion of girls to boys had a borderline statistically significant association with increase violence when we include supportive environment measures in model 5 (Table 2).

**Table 2.** Multi-level logistic regression models presenting associations between student and school-level factors with students' self-reported exposure to physical violence from school staff, in the last week

	Model 1 Null	Model 2 Student factors	Model 3 School structural factors added	Model 4 School academic environment added	Model 5 School supportive environment added
	OR (95% CI), p-value	aOR (95% CI), p-value	aOR (95% CI), p-value	aOR (95% CI), p-value	aOR (95% CI), p-value
Number of observations in model (number of schools)	3706 (42)	3679 (42)	3679 (42)	3679 (42)	3660 (42)
(a) Individual-level student factors					
Female	NA	1.2 (1.0–1.3),	1.2 (1.0–1.3),	1.2 (1.0–1.3),	1.2 (1.0–1.3),
		0.039	0.040	0.038	0.038
Age, years	NA	0.1 (0.9–0.1),	0.1 (0.9–0.1),	0.1 (0.9–0.1),	0.1 (0.9–0.1),
		0.003	0.004	0.004	0.003
Disability	NA	0.9 (0.7–1.2),	0.9 (0.7–1.2),	0.9 (0.7–1.2),	0.9 (0.7–1.2),
		NS	NS	NS	NS
Socioeconomic status					
Amount of time student reports working outside of school					
<1 hour		1	1	1	1
1–2 hours	NA	1.3 (1.2–1.6),	1.4 (1.2–1.6),	1.4 (1.2–1.6),	1.34 (1.2–1.6),
		<0.001	<0.001	<0.001	<0.001
>2 hours	NA	1.7 (1.4–2.1),	1.7 (1.4–2.1),	1.7 (1.4–2.04),	1.7 (1.4–2.1),
		<0.001	<0.001	<0.001	<0.001
More than 3 meals eaten yesterday	NA	0.9 (0.8–1.0),	0.9 (0.8–0.1),	0.9 (0.8–0.1),	0.9 (0.8–1.0),
		0.043	0.034	0.040	NS
Child shared sleeping space with 2 or more other children	NA	1.0 (0.9–1.1), NS	1.0 (0.8–1.1), NS	1.0 (0.8–1.1), NS	1.0 (0.8–1.1), NS
Child shared sleeping space with 1 or more adults	NA	1.1 (0.9–1.3), NS	1.1 (0.9–1.3), NS	1.1 (0.9–1.3), NS	1.1 (0.9–1.3), NS
Boarding at school	NA	NA	1.15 (0.8–1.6),	1.15 (0.9–1.6),	1.15 (0.8–1.6),
			NS	NS	NS
Low performer on educational test	NA	NA	NA	1.14 (0.9–1.4),	1.13 (0.9–1.4),
				NS	NS
Student school connectedness score	NA	NA	NA	NA	0.98 (0.95–1.01),
					NS
(b) School-level factors					
School structural factors					
Urban vs rural	NA	NA	1.2 (0.7–2.0),	1.2 (0.7–2.0),	1.2 (0.7–1.9),
			NS	NS	NS
Boarding vs non-boarding	NA	NA	1.2 (0.7–2.0),	1.2 (0.7–2.1),	1.3 (0.79–2.2),
			NS	NS	NS
Private vs government	NA	NA	1.5 (0.7–3.0),	1.5 (0.7–3.0),	1.2 (0.7–2.4),
			NS	NS	NS
Large vs small	NA	NA	0.9 (0.5–1.6),	0.9 (0.5–1.6),	0.8 (0.5–1.3),
			NS	NS	NS
Male to female ratio within schools					
About equal			1	1	1
>60% girls	NA	NA	1.6 (0.9–2.8),	1.6 (0.9–2.9),	1.7 (1.0–3.0),
			NS	NS	0.048
>60% boys	NA	NA	1.3 (0.6–3.1),	1.3 (0.6–3.1),	1.4 (0.7–3.1),
			NS	NS	NS
Average number of students to teacher in classroom					
Medium (30–44)	NA	NA	1	1	1
Small (12–30)	NA	NA	0.8 (0.5–1.3),	0.8 (0.5–1.3),	0.8 (0.5–1.2),
			NS	NS	NS
Large (45–95)	NA	NA	0.8 (0.5–1.2),	0.8 (0.5–1.2),	1.0 (0.6–1.3),
			NS	NS	NS
Proportion of staff that know of a school written policy on corporal punishment	NA	NA	0.7 (0.3–1.3), NS	0.7 (0.3–1.4), NS	0.6 (0.3–1.2), NS
School social-economic measures					
low vs high proportion of					
Children had three or more meals yesterday	NA	NA	1.0 (0.7–1.5), NS	1.0 (0.7–1.5), NS	1.1 (0.8–1.5), NS
Children shared sleeping area with two or more other children	NA	NA	1.0 (0.7–1.4), NS	1.0 (0.7–1.4), NS	0.94 (0.7–1.3), NS
Children shared sleeping space with one or more adults	NA	NA	0.9 (0.6–1.2), NS	0.9 (0.6–1.2), NS	0.8 (0.6–1.1), NS
Amount of time student reports working outside of school	NA	NA	1.3 (0.8–1.9), NS	1.3 (0.8–1.9), NS	1.0 (0.7–1.6), NS
Academic environment					
Low education performance	NA	NA	NA	1.0 (0.7–1.4),	0.9 (0.7–1.3),
				NS	NS
High ‘academic push’	NA	NA	NA	1.1 (0.7–1.7),	1.0 (0.7–1.6),
				NS	NS
School supportive environment					
High level of ‘student school connectedness’	NA	NA	NA	NA	0.6 (0.5–0.9) 0.003
High level of ‘staff satisfaction with school environment’	NA	NA	NA	NA	1.1 (0.8–1.5) NS
School level general effects					
Variance (SE)	0.2 (0.06)	0.2 (0.06)	0.2 (0.05)	0.2 (0.05)	0.12 (0.04)
VPC %^a^	6.2	6.3	4.6	4.6	3.4
Proportional change in variance^b^	NA	NA	27.9%	0.6%	18.8%

aOR: adjusted odds ratio; NA: not applicable; NS: not significant OR: odds ratio; SE: standard error.

Model 1: null model; model 2: student factors; model 3: school structural factors added; model 4: school academic environment added; model 5: school supportive environment added.

^a^ Variation Partition Coefficient (VPC) see formula 1 shown in Supplementary file.

^b^ Proportional change in variance see formula 2 shown in Supplementary file.

### Between-school variance explained by school-level factors

Our models show that 28% of the between-school variance explained by school-level factors investigated was explained by school structural factors, and less than 1% by academic environment and school-level supportive environment explained 19% proportional change in variance. However, with all the school-level factors in the model, 3.4% of the total variance due to school-level factors remained unexplained.

## Discussion

Our findings suggest that schools with high levels of student connectedness (that is, a collective sense of wellbeing in the school environment) have lower levels of violence from school staff. Other school-level factors were not related to students' risk; instead, in our sample, most variation in risk was due to individual level factors.

Few analyses have explored effects of school-level variables on violence from school staff; hence, we cannot compare our results directly to other literature. However, other studies reported between-school variance accounting for 0.6–2% of the variance in bullying victimisation^30^ and for health outcomes 0–10% of variation was attributable to school-level factors.^9^

Evidence from U.S. National Longitudinal Study on Adolescent Health showed perceived student school connectedness was protective health risk behaviours, including involvement in violence.^2^ One study showed that less victimisation from peers in schools was associated with a better ‘school climate’, including having a policy against violence, positive teacher-student relationship and student participation in decision making.^16^ Another study showed a good school climate, higher socioeconomic status and urban location had a positive effect on pupil outcomes, including wellbeing, problem behaviour and school achievement.^31^

While our findings suggest that the school supportive environment is strongly associated with violence exposure in schools, this could be either a cause or consequence of violence exposure. It may be that students feel more connected to school when staff use less violence, or that students that are more connected behave better and staff use less physical discipline against them. Other influences in students' lives may play an important role—for example, a poor home environment might increase students' feelings of belonging at school.

Contrary to our hypotheses, most other school-level factors investigated during this analysis were not associated with levels of violence against students. The widespread use of physically violent discipline methods in schools with relatively little variation between schools may explain why no school structural factors that we explored had any significant association with student exposure to violence. It could be that between-school differences in the level of violence attributable to the school structural and environmental factors we explored might be detectible in environments where the shift from widespread use of corporal punishment to alternative positive discipline approaches has taken place.

Our findings also suggest that other unobserved school characteristics can influence student's likelihood of experiencing violence from school staff. Our models indicate that school-level factors explain about 6% of students' variation in experience of violence, but school-level variables we investigated left a residual 3.6% of variation attributable to unexplained school differences. It would be useful to consider and investigate other school contextual factors that might explain school-level variation in violence experienced by students in our population.

Being female and younger in age slightly increased the odds of experiencing past week physical violence from school staff. Boys in Israel, Kenya and Ethiopia reported more physical violence from staff compared to girls;^4–6,32^ however, in Tanzania and Zanzibar more girls than boys reported physical violence from a teacher during childhood in a national survey.^33^ Our observation of girls, and students in a school with higher proportion of girls, being more at risk of violence might be due to gender differences in reporting or because girls are more victimised by staff—this finding should be explored further. Working outside of school was associated with a significant increase in levels of past week violence, indicating that students who are doing paid or unpaid employment are more likely to be victimised. These students may need to work to pay school fees or to otherwise help support their family, but might be more likely to be late, absent or have greater difficulty concentrating in class and, therefore, subjected more to physical discipline. Students that reported having eaten three or more meals in the previous day were associated with decreased odds of violence, also suggesting that students with lower socioeconomic status might be at more risk of violence.

### Strengths and limitations

This study is one of a few analyses attempting to quantify the relative importance of school context on, and examine school-level factors associated with, student's experience of violence from school staff in a low resource setting. Our school sample contained a small number of urban schools and private schools, which may have influenced our ability to detect meaningful differences between these types of schools. Our socioeconomic status measures may have been less accurate than using others forms of socioeconomic data at the community level. Our measures for school supportive environment, although shown to have a strong internal reliability in this sample, may not capture all dimensions in the context of Ugandan schools. It might be that experiencing violence is associated with leaving school or not attending school, so those students who were absent during our survey might be at higher risk and our results should not be interpreted as generalisable to them.

### Implications

Examining both the school-level and individual-level factors associated with students' experience of violence from school staff is essential to inform successful intervention development, for both violence prevention and response. This type of analysis can both help target interventions to specific school structural types and highlight school environmental factors that may warrant further research, with the aim of better understanding ways to maintain healthy school environments.

Over half of all students experienced physical violence from staff in the last week. The high level of variation in students' outcomes, which was due to their individual characteristics versus school factors, suggests that the use of physical discipline is highly normalised across schools. In other words, staff in all types of schools with a range of different school-level characteristics are likely to use physical violence against students; but also students are differentially victimised to some degree based on their characteristics. This suggests that interventions to address violence from school staff in this context and similar contexts may benefit from focusing on staff behaviour at the school-level and from exploring why certain students are receiving punishment more often. The Good Schools Toolkit is one such intervention (tested in the trial that this baseline data is drawn from). The Toolkit reduced violence from staff towards students by 42%; however, even with this large reduction, 31% of students in the intervention schools had experienced physical violence from staff in the last week. The intervention was also effective in increasing student individual feelings of ‘safety and wellbeing.’^20^

### Conclusion

In this context, where the prevalence of school violence is very high and there is little variation between schools, school structural factors may be less important determinants of physical violence from school staff towards children. Our analyses do suggest that student school connectedness is associated with violence exposure and that additional individual-level factors are important risks.

## Supplementary data

Supplementary data are available at International Health online (http://inthealth.oxfordjournals.org).

Supplementary Data
