# Supplementary material for: Are school-level factors associated with primary school students' experience of physical violence from school staff in Uganda?
Source: Int Health. 2015 Dec 8;8(1):27–35. doi: 10.1093/inthealth/ihv069 (PMC4716800; doi:10.1093/inthealth/ihv069)
Supplement: Supplementary Data [file supp_8_1_27__index.html]

Are school-level factors associated with primary school students' experience of physical violence from school staff in Uganda? — Are school-level factors associated with primary school students' experience of physical violence from school staff in Uganda? — Supplementary Data 

# Are school-level factors associated with primary school students' experience of physical violence from school staff in Uganda?

## Supplementary Data

Supplementary Data

- Supplementary Data - Docx file
