# Supplementary material for: Are school-level factors associated with primary school students' experience of physical violence from school staff in Uganda?
Source: Int Health. 2015 Dec 8;8(1):27–35. doi: 10.1093/inthealth/ihv069 (PMC4716800; doi:10.1093/inthealth/ihv069)
Supplement: Supplementary Data [file supp_ihv069_ihv069supp.docx]

**Appendix 1.** Formula used for Variation Partition Coefficient (VPC) and proportional change in variance

**Formula 1:** Variation Partition Coefficient (VPC):^1^

**
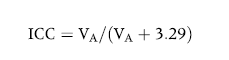
**

Note: VA is the area variance (between-school variance). ICC =intra-class coefficient.

Taken from Merlo, Chaix, Ohlsson et al, 2006, formula 5, page 292^1^

Stata code used in analysis: VPC= VA /( VA +3.29)

**Formula 2:** Proportional change in variance:^1^

**
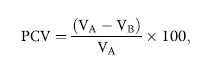
**

Note: VA is the area variance (between-school variance) in initial model and VB is the area variance (between-school variance) in the model with more term.

Stata code used in analysis: PCV= (( VA - VB)/ VA)*100

Taken from Merlo, Chaix, Ohlsson et al, 2006, formula 10, page 297^1^

**References**

1. Merlo J, Chaix B, Ohlsson H et al. A brief conceptual tutorial of multilevel analysis in social epidemiology: using measures of clustering in multilevel logistic regression to investigate contextual phenomena. J Epidemiol Community Health 2006;60:290−7.

**Appendix 2.** Definitions of outcome and composite measures

| **Variable name** | **Instrument, Items** | **Coding** |
| --- | --- | --- |
|  |  |  |
| **Outcome** |  |  |
| Physical violence, self-reported by students  Time frame: past week | International Society for the Prevention of Child Abuse and Neglect Screening Tool-Child Institutional (ICAST-CI).  Assessed via 24 items. Response options included: ‘Yes’ and ‘No’.  Has a school staff member: hurt you or caused pain to you? Slapped you with a hand on your face or head as punishment? Slapped you with a hand on your arm or hand? Twisted your ear as punishment? Twisted your arm as punishment? Pulled your hair as punishment? Hit you by throwing an object at you? Hit you with a closed fist? Hit you with a stick? Caned you? Kicked you? Knocked you on the head as punishment? Made you dig, slash a field, or do other labour as punishment? Hit your fingers or hands with an object as punishment? Crushed your fingers or hands as punishment? Made you stand/kneel in a way that hurts to punish you? Made you stay outside for example in the heat or rain to punish you? Burnt you as punishment? Taken your food away from you as punishment? Forced you to do something that was dangerous? Choked you? Tied you up with a rope or belt at school? Tried to cut you purposefully with a sharp object? Severely beat you up? | Coded 1 if answered yes to any of the items; 0 if answered no to all items. |
| **Individual level** |  |  |
| Student school connectedness | Assessed via 4 items. Response options included: ‘all the time,’ ‘most of the time,’ ‘sometimes,’ ‘never’:   1. I feel that my teachers care about me. 2. I feel safe in school. 3. I feel like I belong at school. 4. I like to spend time at school. | Scores summed, modelled as a continuous variable. Range 0 (low) to 12 (high). |
| **School-level** |  |  |
| School-level student school connectedness | Same as student level connectedness measures (see above). | The school means calculated and schools dichotomised into high or low at 50%. |
| School staff satisfaction with school environment | Assessed via 14 items. Response options included: ‘all the time,’ ‘most of the time,’ ‘sometimes,’ ‘never’:  Job satisfaction:   1. How often would you say you feel that you enjoy your job? 2. Do you feel adequately rewarded financially for what you do? 3. Do you feel valued as an employee? 4. Do you take pride in your work?   General feelings about staff and students:   1. Do you feel that students respect their peers and adults? 2. Do you feel that school staff respect their students? 3. Do you feel concerned about how other school staff members behave at school?   Involvement:   1. In your opinion, do you have enough opportunities to say what you think and contribute to how the school is run? 2. Do you feel that your views on how the school’s policies could be improved are welcomed? 3. How often do you take any actions to change how your school is run? 4. Do you have regular staff meetings?   Support:   1. Do you feel that there is anybody at your school you can talk to if you feel unhappy about work? 2. Thinking about your school as a whole, do you feel like you are part of a team? 3. Do you feel that your employers care about your wellbeing? | Scores summed, modelled as a continuous variable at the staff individual level. Range 0 (low) to 42 (high) for individuals. Then school means calculated and schools dichotomised into high or low at 50%. |
